# Fat Grafting and Adipose Stem Cells for Facial Systemic Sclerosis: A Systematic Review of the Literature

**DOI:** 10.1093/asj/sjae200

**Published:** 2024-09-26

**Authors:** Aurora Almadori, Sze Ching Fung, Christopher P Denton, Peter E M Butler

## Abstract

**Background:**

Orofacial modifications occurring in systemic sclerosis are detrimental for patients, but the therapeutic options are limited.

**Objectives:**

This systematic review aimed to perform an up-to-date appraisal of the literature focusing on fat grafting and other adipose stem cell–based therapies for the treatment of facial systemic sclerosis, determining its efficacy and safety, and investigating the current practice for treatment optimization.

**Methods:**

The review was prospectively registered in PROSPERO (CRD42021286268) and followed the PRISMA principles. Multiple databases were searched and only original studies were included.

**Results:**

Over the 12 studies matching the inclusion criteria, 174 patients were treated. Of these, 87.3% (n = 152) were considered to have improved. The complications, graded with the Clavien-Dindo grading system, were Grade 1 (no treatment required) or Grade 2 (antibiotic required). Patients received a mean [standard deviation] of 2.5 [3.68] (median, 1.35; range, 1-14) lipotransfer procedures. Overall, an average volume of 14.60 [6.24] mL was injected in the facial area (median, 16 mL; range, 3-27 mL). The average interval between procedures was 5.30 [2.04] months (median, 6 months; range 3-6.91 months). At the time of inclusion, patients were diagnosed with scleroderma disease on average after 14.7 [7.35] years.

**Conclusions:**

Fat grafting for facial systemic sclerosis is effective and safe. The definitive durability of the effect is still unclear, and the optimal number of treatments must be determined to define a precise evidence-based protocol. The body of evidence is highly fragmented, with disagreements over surgical techniques and outcome assessments, making results from different studies often not comparable. The level of evidence is overall low or very low, and the risk of bias of published studies is overall medium to high. Randomized controlled trials are urgently needed.

**Level of Evidence: 3 (Therapeutic):**

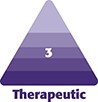

Systemic sclerosis (SSc) is classified into 2 subgroups: limited cutaneous SSc (lcSSc) when the skin fibrosis involves the face and extremities, and diffuse cutaneous SSc (dcSSc) when the skin fibrosis extends on the trunk and proximal portions of the limbs. In both these disease subsets, there is excessive extracellular matrix deposition and reduced remodeling, leading to fibrosis and loss of connective tissue.^1,2^ Irrespective of the disease subset, the signs of the disease are predominantly evident on the face, with a significant impact on facial appearance and function.^1,2^

Typical orofacial features include: subcutaneous tissue loss, fibrotic skin tightly adhered to the underlying planes, reduced facial expression, nasal alar resorption, perioral wrinkles, narrowing of the oral line with decreased mouth opening (microstomia), thinned lips (microcheilia), and dry mouth (xerostomia). These alterations can have a detrimental impact on patients’ psychological well-being and overall quality of life.

The orofacial alterations associated with SSc are the most challenging aspect to correct, and the therapeutic options to address the orofacial fibrosis associated with SSc are limited. Fat grafting and adipose-derived stem cells (ASCs) represent a minimally invasive surgical technique widely used in plastic surgery to increase subcutaneous volumes and ameliorate the skin fibrosis and scarring in multiple conditions, including scleroderma.^3^

The objective of this systematic review was to perform an up-to-date appraisal of the available data in the literature focusing on fat grafting and other ASC-based therapies for the treatment of facial SSc to determine its efficacy and safety, and to investigate the current practice for treatment optimization.

## METHODS

The systematic review was prospectively registered in the PROSPERO database (CRD42021286268) and followed the principles of the Preferred Reporting Items for Systematic Review and Meta-Analyses Protocols (PRISMA-P) statement (www.prisma-statement.org).^4^

### Search Strategy

With the support of a professional academic librarian, a literature search was conducted by A.A. on Embase, MEDLINE, Web of Science, Scopus, Google Scholar, SciELO, The Cochrane Library, and ClinicalTrials.gov in May 2023. Free keywords and MeSH headings related to scleroderma and ASC-based therapies were combined with Boolean operators “and/or”: “scleroderma,” “systemic sclerosis,” “SSc,” “diffuse cutaneous,” “limited cutaneous,” “dcSSc,” “lcSSc,” “lipofilling,” “fat grafting,” “fat transfer,” “lipotransfer,” “fat injection,” “stromal vascular fraction,” “SVF,” “adipose derived stem cells”, “ASCs.”

Examples of full Boolean search strategies for Embase and MEDLINE are illustrated in Supplemental Tables 1 and 2, respectively. The references of the included studies were also reviewed for any relevant publications that might not have been captured in the electronic search.

### Study Selection

The search results were reviewed by A.A. and S.C.F. Disagreements were addressed by panel discussion. After excluding duplicates, all identified articles were screened by reading the titles and abstracts. Selected studies were downloaded and included in this review after full-text reading. Table 1 presents the inclusion and exclusion criteria for this study. Only original studies were included, such as randomized controlled trial (RCTs), case-control studies, cohort studies, case series, and case reports. All surgical techniques used to process the adipose tissue were considered. Reports without original data, such as reviews, discussions, viewpoints, editorials, conference papers, and letters to the editors, were not included. The search was not limited by language, and when required, Google Translator (Google, Mountain View, CA) was adopted.

**Table 1.** Inclusion and Exclusion Criteria

Inclusion criteria	Exclusion criteria
Original studies involving humans in which ASC-based therapy was used to treat a form of facial systemic sclerosis	Nonoriginal studies (review, comment, letter, note, conference paper)
Clinical trials, prospective, retrospective, comparative studies, case series, case reports	Abstract not available/inaccessible in full text
Animal studies assessing the mechanism of action using human-derived ASC-based therapy	Animal studies assessing the mechanism of action using nonhuman-derived ASC-based therapy
Publication in English, Spanish, French, Italian, German, Portuguese or translatable	Localized scleroderma and systemic sclerosis of other areas than the face

ASC, adipose-derived stem cell.

### Data Extraction and Analysis

The full text of the selected papers was read, and their references were checked to retrieve potential papers missed via electronic search. Data from the included studies were recorded and categorized in a Microsoft Excel spreadsheet (Microsoft, Redmond, WA) as follows: (1) study details: first author, year of publication, country; (2) participant demographics: age, gender, disease subset, disease duration, BMI; (3) information regarding the fat grafting procedure: number of treatments, amount injected per session (mL), donor site, recipient site, recipient site preparation (if applicable), fat harvesting method, fat processing method, fat injection method, length of follow-up, and loss to follow-up; (4) efficacy: percentage and number of patients with reported improvement; (5) complications: description of reported complications, percentage and number, and grade according to the Clavien-Dindo classification;^5^ (6) outcome measures: qualitative/quantitative outcome assessment, physician-based outcome measure (validated/nonvalidated), and patient-based outcome measure (validated/nonvalidated).

### Study Outcomes

The primary outcomes of this study were: (1) to determine the efficacy of treatment, defined as the improvement of signs and symptoms, functionality, and patients’ quality of life, as reported with qualitative/quantitative means, by both physicians and patients; and (2) to determine the safety of the treatment, defined as the incidence of intra-/postoperative complications such as infection, oil cysts, nodules, necrosis, and others.

Secondary outcomes were: (1) to determine the optimal treatment modality: when to start the treatment with fat grafting, if multiple procedures are required, the ideal number of treatments (defined as the number of procedures performed until a satisfactory outcome is achieved), and the ideal interval between one treatment and another; (2) to determine the optimal surgical technique: if a method of harvesting, processing, and grafting the adipose tissue is more effective, the ideal amount to be injected in each session, if recipient site preparation improves the outcome, and to determine if lipotransfer used in conjunction with other techniques presents an outcome optimization; and (3) to investigate the methodology used to assess the outcome: qualitative/quantitative outcome assessment, physician-based/patient-based outcome measure (questionnaire, scales); validated/nonvalidated tools.

### Statistical Analysis

The results were summarized in a systematic qualitative synthesis and presented as text and tables. Results are reported as descriptive statistics, such as mean [standard deviation] or median (range), with a 95% CI.

## RESULTS

### Search Results

**Figure 1.**
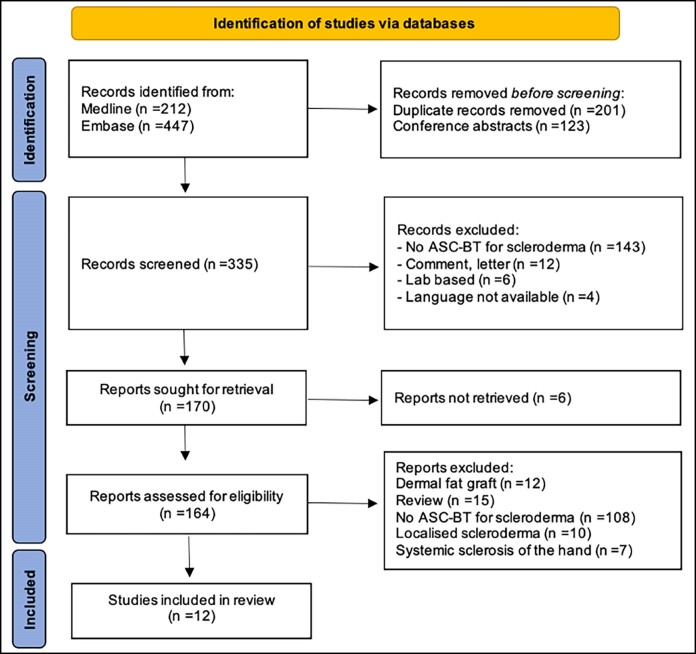
PRISMA flowchart illustrating the screening process to select the papers to be included in this systematic review.

The database search led to the identification of 659 papers (Figure 1). After removing duplicates, a total of 458 abstracts were selected. The reference management software EndNote (Clarivate, Philadelphia, PA) automatically identified 123 conference papers that were excluded. Thus, 335 records were screened for eligibility.

### Study Selection

Two authors (A.A. and S.C.F.) screened the articles. Disagreements were addressed by assessment from a senior author (P.E.B.). The screening process took place in 2 phases: title reading and abstract reading. In total, 165 records that did not meet the inclusion criteria were excluded. The reasons for exclusion were: lack of fat transfer or ASC-based therapy to treat scleroderma (n = 143), letter or comment (n = 12), laboratory-based studies without clinical data (n = 6), language different to the ones mentioned in the inclusion criteria (n = 4).

For the remaining 170 records, download was attempted; of these, 6 were not retrievable. Therefore, 164 articles were downloaded and further screened by full-text reading. Of these, 152 were excluded because they did not meet the inclusion criteria. The reasons for exclusion were as follows: use of dermal fat graft en bloc instead of processing the lipoaspirate (n = 12), narrative review without original results (n = 15), lack of ASC-based therapy to treat scleroderma (n = 108), treatment of localized scleroderma (n = 10), and treatment of SSc of body areas different from the face, ie, hands (n = 7). Therefore, 12 studies were included in this systematic review (Figure 1).

### Data Analysis

The data extracted from the included studies are listed in Supplemental  Tables 2-5.^1,6-16^ The studies were published between 2005 and 2021. The studies were conducted in the following countries: Italy (n = 4), United Kingdom (n = 2), United States (n = 2), France (n = 2), Israel (n = 1), and Iran (n = 1). The study design was mainly prospective (n = 7), followed by retrospective (n = 2), case report (n = 2), and case series (n = 1). The level of evidence ranged between 3 and 5, according to the Oxford Center for Evidence-Based Medicine,^17^ with the majority of studies presenting a level of evidence of 4 (n = 7), followed by level 3 (n = 2) and level 5 (n = 3).

### Demographics

A total of 174 patients were treated (Supplemental Table 3). Of these, 163 were female (93.7%) and 11 males (6.3%). The mean age was 48.89 [10.69] years. BMI was reported in only 2 studies, with an average value of 21.2 [2.26] kg/m^2^. Of the 12 studies, 4 did not specify the type of SSc (28 patients); from the 8 studies specifying the disease subset, 75 patients were affected by dcSSc and 71 by lcSSc. The disease duration prior to treatment was 14.7 [7.35] years. Follow-up was on average 8.73 [4.96] months (median, 9.1 months; range, 2.25-12.41 months).

### Primary Outcome

#### Efficacy

Of the 174 treated patients, 87.3% were considered to have improved (n = 152) (Supplemental Table 4). Due to the high heterogeneity of the methodologies included to assess the outcome, the results were not comparable except for mouth function, which was assessed by determining the Mouth Handicap in Systemic Sclerosis (MHISS) scores in 6 studies, even if actual pre- and postoperative values were provided only in 5 studies. Overall, the average MHISS score reported in these 5 studies was 30.89 [5.74] preoperatively and 22.1 [6.75] postoperatively, with an overall improvement of 8.79 [3.93] points (*P* < .001).

#### Safety

Out of 12 studies, 6 reported on the complications occurring after surgery. The most frequently reported complications were bruising, pain, and swelling of the donor site (4 studies), followed by bruising (2 studies), edema (1 study), and infection of the recipient site (1 study). Only 3 studies reported the number of patients presenting complications: in 1 study, infection occurred and required antibiotic administration in 1 patient out of 62 (1.6%)^1^; in 2 studies, bruising of the donor site was reported in 10 out of 16 patients (62.5%)^9^ and in 1 out of 7 patients (14.2%),^10^ respectively.

The grading of the reported complications was performed according to the Clavien-Dindo grading system.^5^ All reported complications were considered “Grade 1” (no treatment required, resolved spontaneously) except for 1 that was “Grade 2” because antibiotics were required (1 study, 1 patient, 0.57% of overall cases included in the review).

### Secondary Outcomes

#### Treatment Modality

Patients received 1 fat graft in 7 studies, while in 5 studies 2 or more treatments were performed until satisfactory results were achieved (Supplemental Table 5). Overall, patients were offered an average of 2.5 [3.68] fat grafting procedures (median, 1.35; range, 1-14 fat grafting procedures). The average interval between procedures was 5.30 [2.04] months (median, 6 months; range, 3-6.91 months). At the time of inclusion, patients were diagnosed with scleroderma disease after an average of 14.7 [7.35] years.

#### Surgical Technique

The most frequent donor site was the abdominal area (9 studies), followed by the trochanteric area (4 studies), thighs (3 studies), flanks (2 studies), inner knee (1 study), and buttock (1 study) (Supplemental Table 5). In most studies (n = 6), fat was harvested from multiple body areas, rather than just 1 site (n = 5), whereas in 1 study the donor site was not reported.

Donor-site tumescent infiltration was performed in 5 studies and the anesthetics used were: 150 mL of Klein solution (1 study); modified Klein solution with 50 mL saline, 0.5 mL 1:1000 adrenalin, and 10 mL 2% mepivacaine (1 study); modified Klein solution with 100 mL of saline, 20 mL of mepivacaine 2%, 20 mL of ropivacaine 7.5, 1 mL of epinephrine, and 5 mL of sodium bicarbonate solution 1 mEq/mL (1 study); 500 mL of tumescent solution with normal saline, 25 mL lidocaine 2%, and 0.5 mL epinephrine 1:1000 (1 study); 1 liter of sodium chloride 0.9%, 20 mL of lidocaine 2%, and 1 mL of epinephrine 1:200,000 (1 study); 0.5% lidocaine, adrenaline (1:100.000), and 0.8% bicarbonate (1 study). One study adopted the dry technique with no infiltration performed, and 6 studies did not disclose whether donor-site infiltration was performed.

The lipoaspirate was harvested with a blunt 3-mm cannula (4 studies) connected to 10-mL (3 studies) or 60-mL (1 study) syringes; with a 14-gauge cannula connected to a 10-mL syringe (2 studies); and a 10-gauge cannula connected to a 10-mL syringe (1 study); 5 studies did not specify the diameter of the harvesting cannula.

The lipoaspirate was processed by: centrifugation at 3000 rpm for 3 minutes (4 studies), 2700 rpm for 5 minutes (1 study), 2547 rpm for 3 minutes (1 study), decanted by gravity for 10 minutes (2 studies), or 15 minutes (1 study), and washed in a closed 50 mL device (2 studies). One study did not report on the processing method.

The processed lipoaspirate was injected in the facial area with the following tools: 2-mm blunt cannula connected to 1-mL syringe (2 studies); 0.5- to 0.7-mm cannulas (1 study); 15-gauge infiltration cannula (1 study); 17-gauge cannula (1 study); 18-gauge cannula connected to 1-mL syringe (1 study); 19-gauge cannula connected to 2.5-mL syringe (1 study); 21-gauge 0.8-mm cannula (2 studies). In 3 studies, the type of cannula used for injection was not reported.

Overall, an average volume of 14.6 [6.2] mL was injected in the overall facial area (median, 16 mL; range, 3-27 mL). Only in 4 studies were details of the aesthetic units injected provided, as follows: 1 study treated the nose by injecting 1.2 [0.3] mL; 2 studies treated the cheeks with an average of 3.9 [1.8] mL; 1 study treated the chin with 1.9 [0.8] mL; 1 study treated the marionette lines with 2.9 [0.7] mL; 1 study treated the nasolabial fold with 0.9 [0.3] mL; 4 studies treated the oral area by injecting 10 [7.63] mL (median, 8.86 mL; range, 4-16 mL).

Of the 12 studies, 9 involved the injection of adipose tissue alone. One study reported the injection of lab-expanded ASCs at a dose of 8 × 10^5^ suspended in 4 mL of hyaluronic acid.^12^ In 1 study, 10 to 12 cm^3^ of platelet-rich plasma obtained from peripheral blood was injected into the perioral area approximately 10 minutes before fat injection.^13^ In another study, an average of 6.26 mL of platelet-rich plasma was mixed with an average of 19.25 mL of lipoaspirate (microfat) to obtain a final mix, which was injected subdermally and intradermally in different facial aesthetic units.^15^

The operative time was reported in only 1 study with a range of 60 to 90 minutes per operation, while 11 studies did not report the operative time.

Two studies described additional surgical procedures performed before or in combination with fat grafting. These include phenol peel in 1 case report^14^ and a more comprehensive surgical reconstruction in another case report, involving full-thickness skin graft and free buccal mucosal graft to the lower lip, free abdominal mucosal graft with V-Y mucosal advancement flap to lower lip, facial suspension to chin and lower lip, tensor fascia lata graft to chin, mental silicone implant, Z-plasty to lip, V-Y advancement flap to lip, and placement of a mental implant.^7^

### Outcome Assessment

#### Qualitative/Quantitative Objective Assessment

Standard 2-dimensional (2D) photography was adopted in 5 studies; pre- and postoperative photographs were then subjectively graded by physicians using nonvalidated tools. In one study, 3D imaging was used (3dMD Torso System), and volumetric analysis was performed with a designated software (Vultus) to objectively quantify the volumetric retention rate of the implanted fat and its survival over time.^1^

Direct measurement of mouth opening with a digital caliper was performed in 8 studies. These included heterogeneous measurements, such as the maximum interincisal distance (8 studies), distance between the angles of the mouth (2 studies), mouth perimeter (1 study), lip thickness (1 study), and other methods not specified (1 study).

Skin assessment was performed by evaluating different parameters: skin hardness measured with a handheld digital durometer in 1 study; skin elasticity with cutometry (Cutometer Dual MPA 580, Courage & Khazaka Electronic GmbH, Cologne, Germany) in 2 studies; skin elasticity with a nonspecified skin suction elastometer in 1 study; and skin fibrosis (collagen pattern and content) using a Reviscometer in 1 study.

In 2 studies, punch biopsies were performed; one consisted of lip biopsy assessing keratosis and fibrosis, while in the other, the samples were harvested in the lip commissure to assess dermoepidermic junction flattening and microvascular density by counting the absolute number of CD31+ and CD34+ vessels per high-power field.

Vascularization was assessed with videocapillaroscopy of the lower lip using a computerized system called Videocap 200-DS (Medigroup) in 1 study; and with videodermatoscopy of the upper left lip to assess capillary density and vasal ectasia in 1 study.

Mouth dryness, or xerostomia, was assessed in 1 study using the sugar test, which consisted of measuring the time required to melt sugar without crunching it.

#### Physician-Based Assessment

This included mainly nonvalidated grading of clinical 2D photographs (3 studies): 1 showed none/mild/moderate/severe; 1 showed worsening/no improvement/some improvement/much improvement; and a third described the results with a visual analog scale (VAS) from 1 (no improvement) to 10 (maximum possible improvement).

The Modified Rodnan Skin Score (mRSS) was also used to evaluate skin thickness in 2 studies. In 7 studies, a physician-based assessment was mentioned but not further specified.

#### Patient-Based Assessment

Mouth function assessment was included in 7 studies: in 1 study with a nonvalidated VAS for mouth opening (0-100), and in 6 studies it was measured with the validated MHISS score. Of these, only 6 studies reported the pre- and postoperative MHISS score. Overall, the average MHISS score reported in 5 studies was 30.89 [5.74] preoperatively, and 22.1 [6.75] postoperatively, with an overall improvement of 8.79 [3.93] points (*P* < .001).

The majority of included studies (8/12) assessed patient satisfaction, although none of the included tools had been previously validated: 1 study used a 3-point scale on degree of improvement (0, unsatisfied; 1, somewhat satisfied; 2, very satisfied); in 3 studies a 4-point scale on degree of satisfaction was used (unsatisfied, mildly/moderately satisfied, rather satisfied, and very satisfied); 1 study adopted a 10-point scale with 1 being the lowest and 10 being the highest level of satisfaction; in 3 studies details were not provided.

Pain was considered an outcome assessment in 2 studies: in 1 study, it was measured with a VAS and short-form McGill Pain Questionnaire (SF-MPQ), while in the other with a VAS (0-100) on pain induced by the palpation of the masseter and temporal muscles and another VAS (0-100) on facial pain.

In 2 studies, the perception of disability was quantified by means of the validated Health Assessment Questionnaire (HAQ).

Other quality-of-life measures included (in 1 study) were the DAS 24 (satisfaction with appearance), HADS (anxiety and depression), BFNES (preoccupation with other people's judgement), and VAS score (0-10) for perceived noticeability of disfigurement.

Mouth dryness was investigated in only 1 study by means of the validated Xerostomia Inventory Questionnaire and a VAS (0-100) for sicca syndrome.

#### Level of Evidence

The studies were prospective (7/12), retrospective (2/12), case reports (2/12), and case series (1/12). The level of evidence was 4 (7/12), 3 (2/12), and 5 (3/12). The risk of bias was moderate (9/12), serious (1/12), low (1712), and high (1/12) (Supplemental Table 6).

## DISCUSSION

This systematic review of the literature on the use of therapeutic approaches involving ASC-based therapies showed the great potential of these techniques for facial SSc. Considering the overall high effectiveness reported of 87.3% (152/174 patients), fat grafting can be considered a valuable minimally invasive treatment to ameliorate the effects of facial scleroderma. The improvement was particularly evident in the mouth function (*P* < .001 across 5 studies).

The effectiveness reported was also supported by an overall contained complication rate, which was limited mainly to general postsurgical events common to all surgical procedures, such as bruising, edema, and pain. Intervention (nonsurgical) was required in only 0.57% of cases (1/174 patients) for a reported infection, and it was solved with antibiotic administration. Unfortunately, the majority of the studies reviewed did not disclose the percentage or actual number of complications; therefore, an accurate calculation of the complication rate was challenging.

With this surgical technique, multiple facial aesthetic units can be targeted both to enhance the soft tissue bulk and ameliorate fibrosis, improving mouth function and overall skin quality. Among the included studies, the amount of injected fat varied significantly in the oral area: on average the mouth injection was 10 [7.63] mL (range, 4-16 mL). Conversely, the amount injected in the other facial aesthetic units was more consistent among different studies: nose, 1.2 [0.3] mL; cheeks, 3.9 [1.8] mL; chin, 1.9 [0.8] mL; marionette lines, 2.9 [0.7] mL; and nasolabial fold, 0.9 [0.3] mL.

Overall, the combined volumetric and antifibrotic effects exerted on the different aesthetic units of the face contributed to the improvement of mouth function and positively affected the overall quality of life of SSc patients. Across the included studies, this positive effect was attributed mainly to the ASCs; however, ASC-mediated antifibrotic effects are not clearly understood yet. ASCs were extracted, characterized, and implemented in clinical use in only 1 study; however, no statistically significant difference was noted between the ASC group and the fat grafting group.^12^ Further molecular studies are required to understand the mechanism of action of ASCs.

Multiple considerations can be drawn from the results of this systematic review. The first regards the surgical technique adopted, which should be standardized as much as possible because a change in any of the passages of harvesting, processing, or injecting adipose tissue can alter the final by-product, adding variables, and making the surgical outcome less predictable. Among the included studies, we found that only 3 studies adopted the same technique, which was the pure lipostructure technique described by Coleman. The majority of the studies adopted either a modification of the technique (ie, blunt cannula connected to a 60-mL syringe instead of a 10-mL syringe) or a different method (ie, microfat grafting technique or washing).

Another aspect regards the outcome assessment. There is a high variability in the methodology implemented to assess the outcome in studies published so far, making the results not comparable.

A consensus on a core outcome set to assess SSc should be reached, and researchers/doctors carrying out future studies should be encouraged to adopt the same methodology to allow future comparisons and meta-analyses. Volumetric augmentation is one of the main indications for this technique; however, only 1 study assessed this aspect using a validated 3D imaging system. 3D imaging should be implemented as an essential component in future studies to objectively assess the retention rate over time, given that the durability of the effect remains the main unanswered question. Another important aspect is fibrosis improvement, which was assessed only in 3 studies that used different qualitative/quantitative methods (ie, cutometer or histological features). The use of such methodologies should be validated in SSc patients and potentially implemented in future studies. Finally, as microstomia improvement represents one of the main endpoints achievable with this technique, standardization of mouth opening measurement is required. Out of 12 studies, 9 included direct measurement of mouth opening performed with a digital caliper. However, this method does not produce valuable results because it does not consider soft tissue distention, which is improved with soft tissue fat grafting;^18^ therefore, alternative and more effective methods should be used in future studies.

Overall, a more homogeneous methodology in assessing the outcome is advisable in future studies, to allow regression analyses and a better understanding of how to optimize the surgical technique. Outcome assessment should include doctor-based evaluation, patient-reported outcome measures, and objective qualitative/quantitative tools.

Drawing conclusions from the studies published to date to inform clinical practice is still premature for a number of reasons. First, the body of evidence is highly fragmented, with disagreements about the surgical technique used and outcome assessment, making results from different studies often not comparable; the level of evidence is overall low or very low due to the lack of RCTs; and the risk of bias of published studies is overall medium to high.

The use of fat grafting in scleroderma has grown over the last 2 decades because adipose tissue has been proven to be a source of ASCs, with reparative, angiogenic, and immunomodulatory properties.^19,20^ RCTs are urgently needed to rule out a potential placebo effect, which is usually high in cohort studies that mainly use patient-reported outcome measures to assess the outcome.

## CONCLUSIONS

The studies included in this systematic review have reported innovative and effective interventions to correct form and function in facial SSc. However, studies published thus far present limitations, such as small study power, heterogeneous outcome assessment (often implementing nonvalidated tools), and short-term follow-up. The definitive durability of the effect is still unclear, and the optimal number of treatments must be determined to define a precise evidence-based protocol. RCTs are required to confirm these results, and molecular studies are encouraged to clarify the mechanism of action.

## Supplemental Material

This article contains supplemental material located online at https://doi.org/10.1093/asj/sjae200.

## Supplementary Material

sjae200_Supplementary_Data
