# Supplementary material for: Fat Grafting and Adipose Stem Cells for Facial Systemic Sclerosis: A Systematic Review of the Literature
Source: Aesthet Surg J. 2024 Sep 26;45(1):NP25–30. doi: 10.1093/asj/sjae200 (PMC11634384; doi:10.1093/asj/sjae200)
Supplement: sjae200_Supplementary_Data [file sjae200_supplementary_data.zip › Supplemental Table 1.docx]

**Supplemental Table 1. Example of full Boolean search strategy on Ovid MEDLINE**

| 1. scleroderma.mp. or scleroderma, limited/ or scleroderma, diffuse/ or scleroderma, systemic/ or scleroderma, localized/ (26337) |
| --- |
| 1. systemic sclerosis.mp. or scleroderma, systemic/ (22961) |
| 1. scleroderma, limited/ or scleroderma, diffuse/ or scleroderma, systemic/ or scleroderma, localized/ (23669) |
| 1. diffuse cutaneous systemic sclerosis.mp. or scleroderma, diffuse/ (1112) |
| 1. scleroderma, systemic/ (20132) |
| 1. systemic sclerosis.mp. or scleroderma, systemic/ (22961) |
| 1. scleroderma, diffuse/ or scleroderma, systemic/ (20804) |
| 1. scleroderma, systemic/ or scleroderma, limited/ or scleroderma, diffuse/ (20955) |
| 1. 1 or 2 or 3 or 4 or 5 or 6 or 7 or 8 (28529) |
| 1. adipose tissue/ or exp adipose tissue, white/ (95718) |
| 1. transplantation/ or tissue transplantation/ or transplantation, autologous/ (62110) |
| 1. 10 and 11 (1647) |
| 1. (fat or lipo or adipose) adj2 (graft* or transfer* or transplant* or model*)).mp. [mp=title, abstract, original title, name of substance word, subject heading word, floating sub-heading word, keyword heading word, organism supplementary concept word, protocol supplementary concept word, rare disease supplementary concept word, unique identifier, synonyms] (4735) |
| 1. (lipo fill* or lipofill* or lipomodel*).mp. [mp=title, abstract, original title, name of substance word, subject heading word, floating sub-heading word, keyword heading word, organism supplementary concept word, protocol supplementary concept word, rare disease supplementary concept word, unique identifier, synonyms] (471) |
| 1. stem cells/ or adipose tissue/ or stromal cells/ or lipotransfer.mp. or reconstructive surgical procedures/ (209663) |
| 1. 12 or 13 or 14 or 15 (211350) |
| 1. 9 and 16 (212) |
