# Supplementary material for: Fat Grafting and Adipose Stem Cells for Facial Systemic Sclerosis: A Systematic Review of the Literature
Source: Aesthet Surg J. 2024 Sep 26;45(1):NP25–30. doi: 10.1093/asj/sjae200 (PMC11634384; doi:10.1093/asj/sjae200)
Supplement: sjae200_Supplementary_Data [file sjae200_supplementary_data.zip › Supplemental_Table_2.docx]

**Supplemental Table 2. Example of full Boolean search strategy on EMBASE.**

| 1. scleroderma.mp. or scleroderma, limited/ or scleroderma, diffuse/ or scleroderma, systemic/ or scleroderma, localized/ (38078) |
| --- |
| 1. systemic sclerosis.mp. or scleroderma, systemic/ (33070) |
| 1. scleroderma, limited/ or scleroderma, diffuse/ or scleroderma, systemic/ or scleroderma, localized/ (18882) |
| 1. scleroderma, localized/ or scleroderma, systemic/ (17925) |
| 1. diffuse cutaneous systemic sclerosis.mp. or scleroderma, diffuse/ (1444) |
| 1. scleroderma, systemic/ (17165) |
| 1. systemic sclerosis.mp. or scleroderma, systemic/ (33070) |
| 1. scleroderma, systemic/ (17165) |
| 1. scleroderma, diffuse/ or scleroderma, systemic/ (17976) |
| 1. scleroderma, systemic/ or scleroderma, limited/ or scleroderma, diffuse/ (18134) |
| 1. 1 or 2 or 3 or 4 or 5 or 6 or 7 or 8 or 9 or 10 (48932) |
| 1. adipose tissue/ or exp adipose tissue, white/ (86533) |
| 1. transplantation/ or tissue transplantation/ or transplantation, autologous/ (170481) |
| 1. 12 and 13 (2676) |
| 1. (fat or lipo or adipose) adj2 (graft* or transfer* or transplant* or model*)).mp. [mp=title, abstract, heading word, drug trade name, original title, device manufacturer, drug manufacturer, device trade name, keyword, floating subheading word, candidate term word] (7075) |
| 1. (lipo fill* or lipofill* or lipomodel*).mp. [mp=title, abstract, heading word, drug trade name, original title, device manufacturer, drug manufacturer, device trade name, keyword, floating subheading word, candidate term word] (806) |
| 1. stem cells/ or adipose tissue/ or stromal cells/ or lipotransfer.mp. or reconstructive surgical procedures/ (222563) |
| 1. 14 or 15 or 16 or 17 (227330) |
| 1. 11 and 18 (447) |
