# Supplementary material for: Fat Grafting and Adipose Stem Cells for Facial Systemic Sclerosis: A Systematic Review of the Literature
Source: Aesthet Surg J. 2024 Sep 26;45(1):NP25–30. doi: 10.1093/asj/sjae200 (PMC11634384; doi:10.1093/asj/sjae200)
Supplement: sjae200_Supplementary_Data [file sjae200_supplementary_data.zip › Supplemental_Table_3.docx]

**Supplemental Table 3. Demographics information from papers included in this review**

| Author, Year | Country | Age Mean (Sd) or Median (Range) | Gender | BMI | Other Previous Surgery Performed | Time from diagnosis *(years)* |
| --- | --- | --- | --- | --- | --- | --- |
| Almadori,  2019 ^[1]^ | UK | 56 (±11.59) | 61 F,  1 M | NR | NR | 15 (±8.81) |
| Strong,  2021 ^[6]^ | US | 48.7 (± 17.6) | F | NR | NR | 19 (± 12.11) |
| Jeon,  2020 ^[7]^ | UK | 42 year old | F | NR | Full-thickness skin graft to lower lip, free buccal mucosal graft to lower lip, free abdominal mucosal graft with V-Y mucosal advancement flap to lower lip, facial suspension with Mitek to chin and lower lip, tensor fascia lata graft to chin, mental silicon implant, Z-plasty to lip, V-Y advancement flap to lip, replacement of mental implant. | NR |
| Pignatti,  2020 ^[8]^ | Italy | 55.9 (± 9.25) | 19 F,  6 M | NR | NR | 15.39 (± 60.5) |
| Gheisari,  2018 ^[9]^ | Iran | 39 (± 8.32) | F | NR | NR | 7 (±1.79) |
| Blezien 2017 ^[10]^ | US (New York) | 46.28 (± 6.37) | F | NR | NR | 10 (± 4.2) |
| Papa,  2015 ^[11]^ | Italy | 35 (± 15) | F | NR | NR | 11 (± 10) |
| Onesti,  2015 ^[12]^ | Italy | 33 (± 8.46) | 8 F,  2 M | NR | NR | 9 (± 5.45) |
| Virzi,  2017 ^[13]^ | Italy | 41 to 63 | 3 F,  2 M | NR | NR | 9 (± 7.09) |
| Ramon,  2005 ^[14]^ | Israel | 64 | F | NR | Deep phenol peel | 20 |
| Philandrianos,  2017 ^[15]^ | France | 58 and 64 | F | 19.2 and 22.9 | NR | NR |
| Sauterau,  2016 ^[16]^ | France | 53.8 (±9.6) | F | 22.6 (±2.4) | NR | 9.4 (± 6.7) |

***Abbreviations:***

***NR*** *Not reported*

***F*** *Female*

***M*** *Male*
