# Supplementary material for: Fat Grafting and Adipose Stem Cells for Facial Systemic Sclerosis: A Systematic Review of the Literature
Source: Aesthet Surg J. 2024 Sep 26;45(1):NP25–30. doi: 10.1093/asj/sjae200 (PMC11634384; doi:10.1093/asj/sjae200)
Supplement: sjae200_Supplementary_Data [file sjae200_supplementary_data.zip › Supplemental_Table_4.docx]

**Supplemental Table 4. Study details**

| Author, Year | Sample Size | No. of Treatment | Interval Between Treatments (months) | Length of F-Up (months) | Loss to F-Up (% and number) | Patients Improved (% and number) | Complication(s) and % | Complication(s) Grade | Qualitative / Quantitative Outcome Assessment | Physician-Based Outcome Measure(s) | Patient-Based Outcome Measure(s) |
| --- | --- | --- | --- | --- | --- | --- | --- | --- | --- | --- | --- |
| Almadori,  2019 ^[1]^ | 62 | 2,96 | NR | 12,41 | NR | 100% | Infection recepient site wound, treated and solved with antibiotic  1 patient (1.61%) | 2 | a) 3dmd imaging (3Dmd and Vultus software);  b) 2D standard photography;  c) in-vitro analysis | 2D photograph evaluation | a) MHISS (mouth function); b) quality of life: DAS 24, HADS, BFNES,VAS |
| Strong,  2021 ^[6]^ | 10 | 1,7 | NR | 6,2 | NR | 100% | Pain, bruising, swelling at donor site and minor bruising at the recipient site | 1 | Photographs | N/A | Qualitative interviews |
| Jeon,  2020 ^[7]^ | 1 | 14 | 6,91 | 2,25 | 0 | 100% | NR | n/a | Mouth openning | N/A | Not reported |
| Pignatti,  2020 ^[8]^ | 25 | 2,41 | 6 | 6 | NR | 88.8% | Perioral ecchymosis | 1 | a) Mouth opening (interincisal)  b) Photographs,  c) Sialometry | Skin sclerosis (modified Rodnan Skin Score) | a) Pain with VAS and SF-MPQ),  b) perception of disability HAQ and MHISS |
| Gheisari,  2018 ^[9]^ | 16 | 1 | NA | 3 | 0 | 80% | Bruising at zone of harvest  10 patients (62.5%) | 1 | a) Mouth opening capacity  b) Skin biophysical properties (Reviscometer to measure changes in the collagen pattern and content).  c) Photographs | a) Skin sclerosis (Rodnan skin score)  b) 2D photograph evaluation | a) mouth function with MHISS  b) Global patients’ satisfaction |
| Blezien 2017 ^[10]^ | 7 | 1 | NA | 12 | 0 | NR | Graft area oedema;  Harvesting site ecchymosis;  Post-operative pain persistent for more than 3 days | 1 | a) labial biopsy samples;  b) photographs;  c) mouth opening | N/A | MHISS |
| Papa,  2015 ^[11]^ | 20 | 1 | NA | 3 | 0 | NR | NR | n/a | a) Skin biopsies  b) Skin hardness with durometer  c) Videocapillaroscopy | N/A | Non validated semiquantitative score on satisfaction with procedure (1 to 5) |
| Onesti,  2015 ^[12]^ | 10 | 2 | 3 | 12 | N/A | 100% | NR | n/a | Mouth opening | a) VAS 1-10 for improvement | a) MHISS  b) Non validated semiquantitative score on satisfaction with procedure (1 to 3) |
| Virzi,  2017 ^[13]^ | 6 | 1 | NA | 6 | 0 | 100% | NR | n/a | a) Mouth opening  b) cutaneous elasticity (elastometer)  c) Vascularization (videodermatoscope) | Non validated clinical observation | Non validated semiquantitative score on satisfaction with procedure (1 to 10) |
| Ramon,  2005 ^[14]^ | 1 | 1 | NA | 18 | 0 | 100% | None | n/a | none | Non validated clinical observation | Non validated score on satisfaction with procedure |
| Philandrianos,  2017 ^[15]^ | 2 | 1 | NA | 12 | 0 | 100% | 0 None | na | Mouth opening | (Non validated clinical observation | a) MHISS  b) Non validated score on satisfaction with procedure |
| Sauterau,  2016 ^[16]^ | 14 | 1 | NA | 12 | 0 | 91.6% | Bruising  8 patients (57.1%)  Pain  4 patients  (28.5%)  Perioral sensitive manifestation  1 patient (7.14%) | 1 | a) Mouth opening  b) skin elasticity (Cutometer)  c) xerostomia (sugar test) | 2D photograph evaluation | a) Mouth with MHISS and VAS for mouth opening (0-100);  b) Xerostomia with xerostomia Inventory questionnaire and VAS (0–100) for sicca syndrome  c) Pain induced by the palpation of masseters and temporal muscles (VAS 0–100) and facial pain (VAS 0–100)  d) Non validated semiquantitative score on satisfaction with procedure (1 to 4)  e) Global disability: HAQ adapted to SSc |
