# Supplementary material for: Fat Grafting and Adipose Stem Cells for Facial Systemic Sclerosis: A Systematic Review of the Literature
Source: Aesthet Surg J. 2024 Sep 26;45(1):NP25–30. doi: 10.1093/asj/sjae200 (PMC11634384; doi:10.1093/asj/sjae200)
Supplement: sjae200_Supplementary_Data [file sjae200_supplementary_data.zip › Supplemental_Table_5.docx]

**Supplemental Table 5. Details of the surgical procedure**

| Author, Year | Amount Injected | Donor Site | Recipient Site | Comparator Groups | Fat Harvesting Method | Fat Processing Method | Fat Injection Method | Product Used | Operative Time |
| --- | --- | --- | --- | --- | --- | --- | --- | --- | --- |
| Almadori,  2019 ^[1]^ | 10,2 | Abdomen or thighs | face (nose; cheeks; chin; nasolabial folds; upper lip; lower lip) | a) lcSSc versus dcSSc; b) 1-2 treatments versus 3+ treatments; c) immunosuppressed versus not immunosuppressed | Coleman technique - 15 cm x 3 mm disposable cannula connected to a 10cc Luer Lock syringe | Centrifugation at 3000rpm for 3 mins | 1ml Luer-Lock syringes connected to 9 cm by 2mm blunt disposable cannulae | Fat | Not reported |
| Strong,  2021 ^[6]^ | 19,2 | Abdomen or thighs | Face (lips, nasolabial folds, malar regions, marionette lines) | Not reported | 3.0mm liposuction cannula connected 60 ml syringe | Centrifugation at 3000 rpm for 3 minutes | 3ml syringe. 1) face: small incisions were made with 11-blade along lateral commissures and 0.1-0.2 ml aliquots of fat were injected. 2) Hands: small aliquots injected over the dorsum of hand | Fat | Not reported |
| Jeon,  2020 ^[7]^ | 10 | Abdomen | Upper and lower lips, nasolbial folds, cheeks and chin | No comparison | cannula connected to a 10 mL Luer Lock syringe | Centrifugation at 3000 rpm for 3 mins, oil and blood discarded. | Fat injected using cannula connected to 1 ml syringes | Fat | Not reported |
| Pignatti,  2020 ^[8]^ | 16 | Flanks or trochansteric area | Mouth, hands | No comparison | Tumescent infiltration of donor areas with modified Klein solution (50 ml saline solution, 0.5 ml 1:1.000 adrenalin and 10 ml 2% mepivacain) through epidural 23 guage needle attached to a 20 ml Luer-lock syringe.  Harvesting cannulaconnected to 10 ml Luer lock syringe | Centrifugation at 3 mins at 3000 rpm/1900 RCF | 19 gauge needle to introduce a Coleman injection cannula connected to 2.5 ml syringes | Fat | Not reported |
| Gheisari,  2018 ^[9]^ | 27 | Trochanteric area, flank, periumbilical, buttock | perioral, upper lip, lower lip, buccal, malar, periorbital | No comparison | Entry points for the infiltration cannula were anesthetised with 1 ml of pure lidocaine with a 30- gauge needle. Then, 500 ml of tumescent solution (normal saline, 25 ml lidocaine 2%, 0.5 ml epinephrine 1:1000) was infiltrated in the selected donor area with a 1.5 mm cannula.  3 mm blunt cannula connected to 10 ml a Luer-lock syringe | Sedimentation by gravity for 10 minutes. Oil and blood excess were eliminated and the remaining fat was collected. | 1 ml syringe directly injected into the face using disposable 18-gauge cannulas | Fat | Not reported |
| Blezien 2017 ^[10]^ | 3 | Abdomen | Face (lips) | No comparison | Multi-perforated cannulas (around 0.5–0.7 mm). | Sedimentation by gravity for 10 minutes, oil and blood excess eliminated and then platelet rich plasma was added. | Multi-perforated cannulas (around 0.5–0.7 mm) | Fat | Not reported |
| Papa,  2015 ^[11]^ | 12 | Trochanteric area, preiumbilical abdominal region | upper lip, lower lip, mouth corner | No comparison | Tumescent infiltration of 150 ml of local modified Klein solution (containing 100 ml of saline, 20 ml of mepivacaine 2%, 20 ml of ropivacaine 7.5 mg/ml, 1 ml of epinephrine, and 5 ml of sodium bicarbonate solution 1 mEq/ml).  Cannula was connected with a luer-lock syringe | Centrifugation at 700 × g for 3 min | Blunt cannula (Coleman Style II, 9 cm × 17 ga) | Fat | Not reported |
| Onesti,  2015 ^[12]^ | 16 | periumbilical abdominal region | upper and lower perioral region | Fat transplatation and adipose-derived stromal cells - (5 treated with fat, 5 with ASCs) | Local modified Klein solution, 1 liter of sodium chloride 0.9%, 20 mL of lidocaine 2%, and 1 mL of epinephrine 1:200,000 at donor site.  3mm blunt cannula attached to a 10 cc Luer- lock syringe | 1) Fat transplantation: decantation for 15 minutes and only the layer containing adipocytes was used for fat injection;  2) ADSC: Lipoaspirate cell cultivation within 1 hour of isolation. Primary cultures of ADSC expanded following guidelines of current GMP. | 1) Fat transplantation: blunt injection cannula of 2 mm in diameter; 2) ADSC: 2 ml syringe with 30-guage 1/2 needle, cell transferred by 4 ml of hyaluronic acid for each patient | Fat, adipose derived stromal cells | Not reported |
| Virzi,  2017 ^[13]^ | 24 |  | Perioral and malar areas | Adipose-derived mesenchymal stem cells (AD-MSCs) and PRPs | Local infiltration of 150 ml of Klein solution.  10-gauge cannula connected to a 10-ml syringe with luer-loc | Centrifugation for 5 min at 2700 rpm | After 10 min, in the same PRP injection spot, the lipotransfer was performed with a 15-gauge infiltration cannula. Patients remained under observation for 24 hours. | Adipose-derived mesenchymal stem cells | Not reported |
| Ramon,  2005 ^[14]^ | 4 | lower abdomen | perioral area | na | o.5% lidocaine, adrenaline (1:100.000), 0.8% bicarbonate for infiltration | not described. | not described | fat | Not reported |
| Philandrianos,  2017 ^[15]^ | 19,25 | INNER thighs | cheekbones, the nasogenian grooves, superior and inferior lips and the chin | na | lidocaine mixed with NaCl 0.9% solution.  14-Gauge cannula with eight 600 μm orifices from St’rim kit® (Thiebaud Biomedical Device, France). | A closed system was used with anti-return valves, 10 mL syringe and a 50 mL PureGraft® filtration technology device (Pure- graft LLC, USA). Fat was mixed with prp - PRP preparation was performed using the SkinPras® de- vice (Soluciones Biogeneratives, Spain). Briefly, peripheral blood was collected by venipuncture using a 20 mL syringe anticoagulated with 10% ACD-A (9). Blood was transferred in a secondary device and centrifuged (Omnigrafter, Soluciones Biogeneratives) at 3200 rpm over 10 minutes. PRP was col- lected in a 10 mL syringe. A 0.5 mL sample was used for qual- ity controls. | 21-gauge (0.8mm) needle, which was then replaced by a cannula of the same diameter | microfat + prp (26% PRP and 74% microfat containing 53 million of platelets/ mL in case 1 and 30% PRP and 70% microfat containing 17.6 million of platelets/ mL in case 2) | Not reported |
| Sauterau,  2016 ^[16]^ | 16.3 | inner side of the knees, abdomen and hips. |  |  | In local anesthesia entry points for the infiltration cannula were anesthetized with pure 1% adrenaline and lidocaine with a 30-gauge (0.25mm) needle. An infiltration was then carried out in the area with a 14-gauge cannula with 10ml of 1% adrenaline and lidocaine diluted in 20ml of physiological salt solution, with an injected volume at each entry point of 0.5ml.  14-gauge (2-mm cannula height holes of less than 1-mm blunt tip) connected to a 10-ml syringe | closed-circuit PureGraft 50 ml system filtration pocket (Puregraft, San Diego, Calif.). | 21-gauge (0.8mm) needle, which was then replaced by a cannula of the same diameter. | microfat | 60 to 90 minutes |
