# Supplementary material for: Fat Grafting and Adipose Stem Cells for Facial Systemic Sclerosis: A Systematic Review of the Literature
Source: Aesthet Surg J. 2024 Sep 26;45(1):NP25–30. doi: 10.1093/asj/sjae200 (PMC11634384; doi:10.1093/asj/sjae200)
Supplement: sjae200_Supplementary_Data [file sjae200_supplementary_data.zip › Supplemental_Table_6.docx]

**Supplemental Table 6. Level of Evidence**

| Author,  Year | Study Design | Loe | Stenght of Recommendations | Risk Of Bias Rate |
| --- | --- | --- | --- | --- |
| Almadori,  2019 ^[1]^ | Retrospective | 3 | Moderate | Moderate |
| Strong,  2021 [6] | Retrospective | 3 | Low | Serious |
| Jeon,  2020 [7] | Case report | 5 | Moderate | Moderate |
| Pignatti,  2020 [8] | Prospective | 4 | Moderate | Moderate |
| Gheisari,  2018 [9] | Open-label study, prospective | 4 | Moderate | Moderate |
| Blezien 2017 [10] | Prospective | 4 | Low | Low |
| Papa,  2015 [11] | Prospective | 4 | Moderate | Moderate |
| Onesti,  2015 [12] | Prospective | 4 | Moderate | Moderate |
| Virzi,  2017 [13] | Prospective | 4 | Moderate | Moderate |
| Ramon,  2005 [14] | Case report | 5 | low | high |
| Philandrianos,  2017 [15] | case series | 5 | moderate | Moderate |
| Sauterau,  2016 [16] | open-label study (?) | 4 | moderate | Moderate |

## 
